# Recurrence-Free Survival after Synovectomy and Subsequent Radiosynoviorthesis in Patients with Synovitis of the Knee—A Retrospective Data Analysis

**DOI:** 10.3390/jcm13020601

**Published:** 2024-01-21

**Authors:** Melanie Schindler, Stephan Puchner, Jan Reinhard, Franziska Leiss, Reinhard Windhager, Richard Lass

**Affiliations:** 1 Department of Orthopedics and Trauma Surgery, Division of Orthopedics, Medical University of Vienna, 1090 Vienna, Austria; stephan.puchner@meduniwien.ac.at (S.P.); reinhard.windhager@meduniwien.ac.at (R.W.); richard.lass@meduniwien.ac.at (R.L.); 2 Department of Trauma Surgery, University Medical Centre Regensburg, 93053 Regensburg, Germany; 3 Department of Orthopedics, University Medical Centre Regensburg, Asklepios Klinikum Bad Abbach, 93077 Bad Abbach, Germany; jan.reinhard@klinik.uni-regensburg.de (J.R.); franziska.leiss@klinik.uni-regensburg.de (F.L.)

**Keywords:** radiosynoviorthesis, synovectomy, synovitis, knee, survival

## Abstract

Background: Persistent knee synovitis leads to joint discomfort, incapacitating inflammation, and functional limitations. The conventional approach has involved surgical procedures to eliminate the actively inflamed synovial membrane. This study aims to investigate the recurrence-free survival and functional outcome after synovectomy and subsequent radiosynoviorthesis (RSO) in patients with knee synovitis. Methods: Thirty-seven knees diagnosed with pigmented villonodular synovitis (PVNS), rheumatoid arthritis (RA), and peripheral spondyloarthritis underwent synovectomy and subsequent RSO between May 2005 and October 2016. The mean age was 34.9 ± 15.1 years, and the mean follow-up period was 84 ± 36.4 months. Clinical outcomes were assessed using the Oxford Knee Score and the presence of swelling and pain at the last follow-up. Recurrence-free survival denotes the duration from synovectomy to surgical re-synovectomy. Results: In general, twelve knees underwent re-synovectomy after a mean follow-up of 34.8 ± 24.9 months. The recurrence-free survival was 83.8% at two years, 71.3% at five years, and 61.7% at ten years. The subgroup analysis revealed recurrence-free survival at two years in 63.6% of patients with PVNS, 86.7% of those with RA, and 100% of individuals with peripheral spondyloarthritis. Conclusions: This study demonstrates that combined therapy for synovitis is an effective approach, significantly improving clinical outcomes.

## 1. Introduction

Chronic synovitis of the knee, characterized by persistent joint pain, debilitating swelling, and functional impairment, presents a substantial challenge for both the patients experiencing the condition and the orthopedic practitioners entrusted with its management. These symptoms commonly manifest in three primary diagnostic categories: pigmented villonodular synovitis (PVNS), rheumatoid arthritis (RA), and peripheral spondyloarthritis.

Pigmented villonodular synovitis, an infrequent and benign condition, primarily targets the synovial tissue, with a predilection for the knee joint [1]. Also recognized as a diffuse tenosynovial giant cell tumor (TGCT) [2,3], it is characterized by its localized occurrence and impact on the synovium. In contrast, RA manifests as a systemic disorder characterized by asymmetrical and erosive synovitis, affecting multiple joints across the body [4]. In no fewer than 65% of individuals diagnosed with RA, there exists an observed incidence of knee joint involvement [5]. Peripheral spondyloarthritis encompasses inflammatory conditions predominantly affecting joints outside the spine, encompassing entities such as ankylosing spondylitis, psoriatic arthritis, arthritis/spondylitis associated with inflammatory bowel disease, and reactive arthritis [6,7,8]. Diagnostic precision relies on magnetic resonance imaging (MRI) scans and biopsy results, with conservative treatments frequently proving inadequate for mitigating symptoms [9,10,11].

Traditionally, surgical interventions comprising either arthroscopic or open synovectomy of the knee have been widely employed, demonstrating efficacy in enhancing joint function and alleviating pain [12]. However, an emerging therapeutic alternative, radiosynoviorthesis (RSO), also known as radionuclide synovectomy, has introduced a novel paradigm in the management of knee synovitis. RSO involves the intra-articular infusion of a radioisotope into the affected joint, aiming to deactivate the inflammatory stimulus originating from the synovial membrane [13]. Its primary indications encompass inflammatory diseases such as PVNS, RA, and peripheral spondyloarthritis [14].

Against this backdrop of evolving therapeutic modalities, the primary objective of our study is to investigate and report the long-term outcomes associated with a two-step treatment approach as follows: an initial surgical synovectomy followed by a subsequent RSO. Our aim is to evaluate the recurrence-free survival rate and measure functional outcomes in patients with chronic synovitis of the knee. By evaluating the effectiveness and durability of this combined treatment modality, we offer valuable insights for clinicians and patients, providing enhanced therapeutic strategies for managing chronic knee synovitis.

## 2. Material and Methods

### 2.1. Patients

A cohort of 45 patients suffering from chronic synovitis of the knee underwent a comprehensive treatment protocol that involved synovectomy followed by a subsequent RSO at an orthopedic university center. The study period included patients treated between May 2005 and October 2016. In a retrospective analysis, all patients were systematically categorized and assessed through an evaluation of the clinical records with a focus on gender, age at the time of surgery, diagnosis, specific surgical procedures, duration of the operation, clinical outcomes, and any subsequent revisions.

Accordingly, this retrospective single-center analysis included 37 knees from 34 patients. Unfortunately, eleven cases had to be excluded from the study due to irregular follow-up appointments. This study group included 15 female and 19 male individuals (22 right and 15 left knees). The mean age of these patients was 34.9 years (range 14.5–77.0 years). The mean body mass index (BMI) among the individuals in this study was 24.5 (range 16.5–46.3). The mean follow-up period was 84 months (range 2–12.8 years).

The indications for synovectomy and RSO primarily included persistent knee swelling and pain, which resisted conventional conservative treatments such as repeated joint fluid aspirations and corticosteroid injections. Moreover, the presence of synovitis, as confirmed by an MRI, was an additional contributing factor.

After performing a comprehensive histological analysis of the synovitis cases, the patients were distributed into three groups according to the following diagnoses:

1. Eleven cases were diagnosed with PVNS (including five women and six men) with a mean age of 34.4 years ± 18.3 years.
2. Fifteen cases were diagnosed with RA (including six women and nine men) with a mean age of 37.6 years ± 14.7 years.
3. Eleven cases were diagnosed with peripheral spondyloarthritis (including three women and eight men) with a mean age of 31.7 years ± 13.0 years.

The synovectomy procedures, including histological examinations, were performed arthroscopically in 19 knees, while 18 knees underwent an open synovectomy. The choice to perform an open synovectomy was based on the identification of synovitis localized specifically within the dorsal compartment of the knee. These particular patients underwent a synovectomy, performing both anterior and posterior approaches. The mean duration of all surgical operations was 91.1 min, with a range of 25.0 min to 235.0 min. The average time for the arthroscopic synovectomy was 84.2 min (range 25.0–185.0 min), whereas the average time for the open synovectomy operations was 98.3 min (range 35.0–235.0 min).

Subsequently, after a mean period of 18 weeks (range 5.4–53.6 weeks), RSO with yttrium-90 citrate was performed (Table 1). Recurrence-free survival describes the time from synovectomy to surgical re-synovectomy.

To assess the clinical progress, we performed the Oxford Knee Score (OKS) and examined the existence of swelling in the knee during the final follow-up. The OKS, a 12-question survey, assessed pain and limitation-related knee function during everyday activities and work [15]. Each question had five options to choose from, rated from 0 to 4, showing different levels of limitations. In this context, zero represents the highest degree of limitation, while four represents the lowest. The maximum score is 48 points.

### 2.2. Statistics

Statistical analysis was conducted using IBM SPSS Statistics 25 (IBM Corp., Armonk, NY, USA). Demographic and clinical characteristics were presented as average values with a standard deviation and percentage (%). To assess the likelihood of re-intervention, a Kaplan–Meier analysis was performed, considering “any surgical re-intervention” as the endpoint. To determine the association between each parameter and recurrence-free survival, an ANOVA test was performed. The relationship between the diagnosis and the OKS was examined using the Mann–Whitney U test. Moreover, the comparison of mean OKS among the demographic and clinical characteristics was conducted using the T-test. Assessing the association between clinical outcomes and each diagnosis involved a chi-square test of independence. A significance level (alpha) of 0.05 was chosen, denoting that a *p*-value below this threshold indicated a statistically significant relationship between the clinical outcome and each diagnosis. The threshold for statistical significance was set at *p* < 0.05.

### 2.3. Ethical Considerations

The study was conducted in accordance with the Declaration of Helsinki and approved by the Ethics Committee of Vienna (protocol numbers 1855/2018 and 11/2018).

## 3. Results

### 3.1. Recurrence-Free Survival Rate

**Figure 1:**
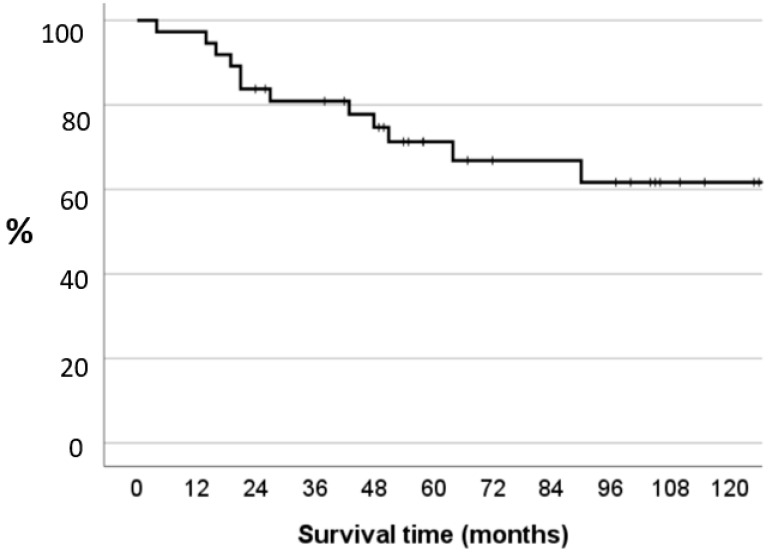
The Kaplan–Meier probability plots of remission of synovitis after operation and yttrium-90 treatment.

In general, 37 knees underwent a synovectomy with a subsequent RSO. Twelve of the 37 knees underwent a re-synovectomy after a mean time of 34.8 months (range 4–90 months) (Figure 1). The recurrence-free survival rate was 83.8% at two years, 71.3% at five years, and 61.7% at ten years. One knee required a total knee arthroplasty after 96 months due to osteoarthritic alterations. No synovectomy had to be performed on this patient. Age, BMI, sex, duration of the operation, and temporal distance between synovectomy and RSO did not influence the recurrence-free survival rate.

**Figure 2:**
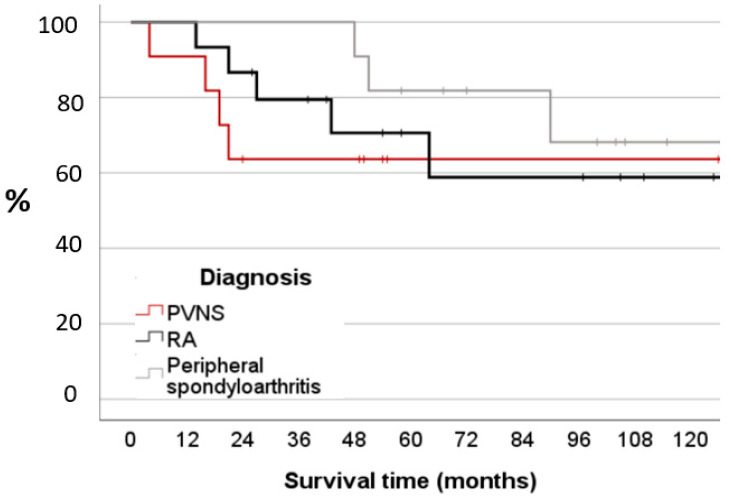
The Kaplan–Meier probability plots of remission of synovitis after operation and yttrium-90 treatment between the different diagnoses.

Out of the eleven cases diagnosed with PVNS, four cases (36%) needed a re-synovectomy after a mean duration of 15.0 ± 7.6 months. Among the fifteen cases in the RA group, five (33%) underwent a re-synovectomy after a mean interval of 33.8 ± 20.0 months. Similarly, in the group with eleven cases diagnosed with peripheral spondyloarthritis, three cases (27%) underwent a re-synovectomy after a mean period of 63.0 ± 23.4 months (Table 1). The subgroup analysis showed 63.6% of the patients with PVNS, 86.7% with RA, and 100% with peripheral spondyloarthritis, with a recurrence-free survival rate at two years and 68.3% at ten years (Figure 2). However, this difference was not significant (*p* = 0.560).

### 3.2. Diagnostic Differences

The patients with a diagnosis of PVNS underwent open synovectomy significantly more frequently compared to other patients (*p* = 0.026) (Table 1). Consequently, the operation duration was also significantly longer compared to the other diagnoses (*p* = 0.038) (Table 1).

The interval between synovectomy and RSO was distributed approximately equally among the three different diagnoses. The follow-up period significantly differed between PVNS and peripheral spondyloarthritis (*p* = 0.23) (Table 1). Among the patients diagnosed with RA, four (27%) received systemic therapies such as methotrexate, cortisone, or sulfasalazine. In the case of peripheral spondyloarthritis, one patient (9%) was treated with sulfasalazine. Out of the total of 37 patients, the majority (32) did not take any medication, while five patients were prescribed medication for their respective conditions.

### 3.3. Functional Outcome

At the final follow-up, the mean Oxford Knee Score (OKS) observed among the patients was 38 points, with a range from 12 to 48 points. Notably, the individuals diagnosed with PVNS achieved a mean OKS of 42.1 ± 4.2 points. Comparatively, the patients diagnosed with RA reached an average OKS of 36.8 ± 9.3 points. Moreover, the patients diagnosed with peripheral spondyloarthritis achieved a mean OKS of 36.9 ± 2.0 points. Despite these variations, the observed differences did not demonstrate statistical significance (PVNS vs. RA, *p* = 0.134; PVNS vs. peripheral spondyloarthritis, *p* = 0.101).

There was no statistical variation observed between the men and women concerning the OKS during the last follow-up assessment (men: 39.1 ± 6.7 points; women: 37.5 ± 8.8 points; *p* = 0.267). The patients who underwent arthroscopic synovectomy achieved an OKS of 36.9 ± 8.8 points, whereas those who underwent open synovectomy attained 40.0 ± 5.8 points. However, the difference was not statistically significant (*p* = 0.109).

A lower OKS was associated with pain during the last follow-up assessment. The patients reporting no pain exhibited a notably higher OKS of 44.7 ± 4.0 points, whereas those experiencing pain had a lower OKS of 35.7 ± 7.2 points (*p* < 0.001). Similar trends were observed concerning swelling, where patients without it demonstrated a higher OKS compared to those with swelling (40.3 ± 5.8 points vs. 35.9 ± 9.1 points). However, this difference did not reach statistical significance (*p* = 0.084). Additionally, the patients who underwent re-synovectomy showed a slightly higher OKS of 39.1 ± 6.5 points compared to those without the procedure (OKS 36.9 ± 9.7 points) and without significance (*p* = 0.208). No statistically significant difference was observed between the OKS and the duration of operations (*p* = 0.381).

The pain was relieved for all patients due to the intervention. Among the recorded cases of PVNS, there were four cases of no pain and seven cases of mild pain, with eleven cases in total. In the RA group, there were also four cases of no pain and eleven cases of mild pain, with fourteen cases in total. Similarly, for peripheral spondyloarthritis, three cases were categorized as no pain, while eight cases were considered mild pain, with eleven cases in total. There was no statistical significance between the various diagnoses (*p* = 0.848). Overall, among the 37 cases evaluated, 11 (30%) had no pain, and 26 (70%) cases were classified as having mild pain at the last follow-up.

At the last follow-up, less than half of the patients (43%) experienced recurring swelling in the operated knee. Specifically, swelling was absent in 73% (*n* = 8) of patients with PVNS, 53% (*n* = 8) of patients with RA, and 46% (*n* = 5) of patients with knees affected by peripheral spondyloarthritis. However, these percentages did not demonstrate statistical significance (*p* = 0.409).

## 4. Discussion

This study represents the inaugural investigation specifically addressing the recurrence-free survival rate after interventions such as synovectomy followed by RSO. The study concentrated on individuals experiencing persistent knee swelling attributable to diverse conditions, including PVNS, RA, and peripheral spondyloarthritis. Contemporary research has demonstrated that the combined implementation of arthroscopic debridement and RSO demonstrates favorable outcomes for individuals with RA, enduring knee swelling, as well as those diagnosed with PVNS [16,17,18,19].

### 4.1. Recurrence-Free Survival Rate

The main focus of our study was on the recurrence-free survival rate, which represents the most critical factor. The survival analysis evaluated that after ten years, approximately 61.7% of patients showed a recurrence-free survival rate. In particular, this study notably observed a trend towards a higher recurrence-free survival rate and better clinical outcomes in patients diagnosed with PVNS and peripheral spondyloarthritis arthritis compared to those diagnosed with RA.

Until now, there have only been a few studies focusing on surgical synovectomy and RSO. There has been a notable lack of research in this area.

Since many studies have revealed a high recurrence rate of PVNS, the combination of both treatments seems to be effective [20,21,22,23]. A review article about PVNS [24] showed better results for surgical debridement combined with radiation synovectomy. Eight studies reported on the results of this combined therapy. The recurrence rate was 25.5% (24 of 96 patients) during a mean follow-up period of 4.5 years. Our survival analysis of PVNS showed that 63.3% (four out of eleven patients) had a recurrence-free survival at 10 years. However, our recurrence rate of 36.7% is higher in this long-term period.

Goetz et al. [16] published the effects of arthroscopic synovectomy and RSO of the knee joint in RA. Moreover, 44% of the patients had re-interventions at ten years and 32% at 14 years. The recurrence-free survival rate was 66% in patients with RA at ten years. These results are similar to the results of our study, which observed a recurrence-free survival rate of 58.8% (five out of 15 patients) in RA at ten years. In a separate study focusing on arthroscopic synovectomy, Tirolo et al. [25] analyzed the survival rates following this specific procedure. Their analysis indicated that 18 out of 66 knees, constituting 27.3% of the cases, needed subsequent revision procedures. These revision surgeries occurred around 48.6 months following the initial arthroscopic synovectomy.

Akmese et al. [17] evaluated the effects of arthroscopic subtotal synovectomy combined with RSO in chronic non-specific synovitis of the knee. At the last follow-up, clinical symptoms such as pain and swelling had improved, and range of motion had decreased. The combination of both treatment strategies seems to be an effective therapy.

The recurrence-free survival rates depended on the underlying primary diagnosis. The progress of the survival curve for the different diagnoses is remarkable. PVNS achieved the maximum recurrence rate during a follow-up of two years, and RA at five years. The patients with peripheral spondyloarthritis had a recurrence-free survival rate of 100% at two years.

### 4.2. Radiosynoviorthesis

Radiosynoviorthesis (RSO) consists of an intra-articular injection of radionuclides into the joint capsule. We used yttrium-90 citrate. It stops the inflammation activity of the synovium and can relieve pain. There are notable disparities in the adoption of radiosynoviorthesis across different regions worldwide. This described method is commonly performed in Europe but is infrequently adopted in the USA, where systemic therapy is generally the initial preference [26]. Nevertheless, standardized protocols for radiosynoviorthesis usage exist in Europe and other global regions. It is crucial to minimize unnecessary radiation exposure for risk groups in nuclear medicine. Advising these particular individuals to stay around 1.0 m away from the patient is recommended. The study by Oliveria et al. highlights the recorded ambient dose equivalent and shows that RSO is a safe procedure for family members [27].

Recent studies have reported on the effects of RSO [28,29]. For instance, Zuderman et al. reported a significantly better response rate in patients with RA than those with osteoarthritis (OA). The reason could be the pathophysiology mechanism being an inflammatory stimulus in RA and a mechanical one in OA [13]. A study by Taylor et al. [30] on RSO in knee arthritis showed an improvement of 46% in knees after 12 months. In a review article on PVNS, a recurrence rate of 55.5% can be found for radiation synovectomy [24]. Dürr et al. compared the results of open synovectomy in PVNS patients with or without RSO. This study describes a reduction in the recurrence-free survival rate after synvectomy with RSO [31]. Substantial evidence suggests that RSO is a beneficial treatment for enduring synovitis, whether it occurs from conditions such as RA, psoriasis arthritis, undefined spondyloarthropathy, osteoarthritis, or other reasons [32,33,34]. Nevertheless, it is important to highlight that the efficacy of RSO might decrease over time with repeated use. In many parts of the world, the complete healing benefits of RSO are not fully exploited or utilized for these specific patients. Furthermore, it is important to emphasize the possible adverse effects associated with RSO, which may include conditions like arthrofibrosis, arthritis, osteonecrosis, thrombosis, wound necrosis, infection, and, in rare instances, radiation-induced sarcoma [2,3,26,35,36]. Side effects or complications after RSO are quite rare, aside from non-serious side effects such as transient radiogenic synovitis with recurrent effusion or a flush from the co-injected glucocorticosteroid. The probability of a seriously adverse event (e.g., intra-articular infection or radiogenic tissue necrosis) is below 0.1 per 1000 [37]. The radiogenic induction of a malignant tumor after RSO has never been described, even after a long-term follow-up [38].

Nevertheless, it is important to emphasize the existence of pharmacological approaches for managing RA and peripheral spondyloarthritis. The current treatment guidelines strongly advocate for the implementation of systemic therapy, which includes the use of antimetabolites and/or biologics to effectively tackle active diseases in individuals with RA and peripheral spondyloarthritis [39,40]. This systemic therapy approach is recommended to address the underlying disease activity associated with both RA and peripheral spondyloarthritis, acknowledging that these conditions are not solely confined to joint-related issues but also affect the body systemically. The utilization of antimetabolites and biologics has shown promising outcomes in managing the disease activity and symptoms associated with these conditions, presenting a beneficial algorithm for patients seeking effective long-term management strategies.

Alongside the risks of RSO, it is worth noting that systemic medications such as tyrosine kinase inhibitors (such as nilotinib and imatinib) or drugs that target the colony-stimulating factor-1 (CSF-1) pathway (such as pexidartinib and emactuzumab) have demonstrated effectiveness against the TGCT and might offer alternative treatment options in specific situations [41,42]. Tyrosine kinase-inhibitors (TKI), including Spleen tyrosine kinase (SYK) (fostamatinib) and Janus kinases (tofacitinib), are two novel oral therapies that have demonstrated good short-term clinical responses in active rheumatoid arthritis patients [43]. In the TGCT, a neoplastic clone constitutes a subpopulation (2–16%) [44] of cells that overexpress CSF-1. Inhibition of CSF1/CSF-1 receptor (CSF-1R) signaling has shown efficacy in the treatment of locally advanced and recurrent diffuse TGCT [45,46,47]. Verspoor et al. evaluated the long-term efficacy of imatinib mesylate in patients with an advanced TGCT. Moreover, 17 of the 58 evaluable patients achieved a complete response (CR) or partial response (PR). One year and five year progression-free survival rates were 71% and 48%, respectively [41].

### 4.3. Differences between Diagnoses

The diagnosis of PVNS underwent a notably higher frequency of open synovectomy compared to the other diagnoses. This concept has been well documented in the existing literature. The utilization of both anterior and posterior open synovectomyperformed under direct visualization, has shown recurrence rates comparable to the best-reported rates for this condition [48,49].

Over the last few years, arthroscopic synovectomy for the therapy of chronic synovial swelling of the knee joint has become an alternative treatment to open synovectomy. Several studies have proven arthroscopic synovectomy as an effective therapy [12,18,24,50,51]. On the one hand, arthroscopic synovectomy is less invasive, requires shorter hospitalization, and allows early mobilization and minimal loss of motion [12,30]. On the other hand, open synovectomy requires a wide skin incision and has a higher infection rate. However, it demonstrates a lower recurrence rate of synovitis due to the more thorough removal of the synovial tissue [52]. We did not detect significant differences between the arthroscopic or open synovectomy concerning the recurrence-free survival rate. Other studies showed similar results [12,18,24,50]. 

A study conducted by Ossyssed et al. [53] incorporated eight non-inflamed control subjects and eight RA patients utilizing a two-stage synovectomy methodology. A significant proportion of the patient cohort (94%) exhibited sensory denervation subsequent to surgical synovectomy, concomitant with a cellular response associated with wound healing. The findings of this investigation contribute to elucidating the favorable outcomes associated with surgical synovectomy, commonly characterized by diminished pain and enhanced mobility.

### 4.4. Functional Outcome

Presently, there are few studies that specifically focus on the clinical outcome following synovectomy and subsequent RSO in detail. Most studies focus on the surgical procedure or only the RSO without considering both. In our study, the pain was relieved in all patients due to the intervention. The study conducted by Klug S. et al. [52] showed a significant improvement in pain in patients with RA who underwent arthroscopic and radiation synovectomy. Hufeland et al. [54] concentrated on the long-term outcomes of PVNS affecting the hip after synovectomy and RSO. They found a good clinical outcome with a mean modified Harris Hip Score of 91 points.

The following studies focus on the outcomes after RSO. A multicenter study assessed the primary outcome using a composite change index (CCI) [55]. The CCI included various aspects such as functional disability, pain measured on a Visual Analogue Scale, knee tenderness, swelling, effusion, and the overall therapy assessment by both the patient and physician. Effective therapy was defined as a CCI score of six or higher. This evaluation occurred six months post-therapy, following the 90Y lag phase. The study concluded that the spread of intra-articular 90Y did not impact the clinical effectiveness of knee RSO. In contrast, Liepe [56] evaluated 58 knees and observed an excellent or good response in 57% of the treated knees. It indicates the absence of symptoms or a significant reduction in symptoms.

### 4.5. Limitations

In the context of this investigation, it is imperative to acknowledge several inherent limitations. One primary constraint lies in the retrospective nature of the study. The retrospective design inherently introduces constraints on data collection and analysis, which may compromise the depth and accuracy of the findings. Specifically, within the study cohort comprising 22 patients, only five individuals were identified as having undergone systemic therapy. Systemic therapy is a critical aspect of managing both RA and peripheral spondyloarthritis. The absence of information about this treatment in the study might result in significant missing data, which could substantially impact the overall findings.

The retrospective orientation of the study design not only restricts the identification of patients receiving systemic therapy but also raises concerns regarding the availability of comprehensive information on this therapeutic intervention. The lack of data pertaining to systemic therapy introduces a notable gap in the study’s dataset, potentially resulting in substantial missing information that could significantly influence the overall outcome and interpretation of the findings.

Furthermore, another noteworthy limitation pertains to the relatively diminutive size of the study cohort. The involvement of a limited number of patients imposes challenges in extrapolating broad and conclusive inferences from the collected data. Specifically, within the study cohort consisting of 22 patients, only five individuals were identified as having undergone systemic therapy—a crucial component in the management of RA and peripheral spondyloarthritis. Moreover, the ten-year follow-up only included 61% of the patients and the attrition of eleven patients during this period, constituting approximately 30% of the initial patient pool, which further compounds the challenge of drawing robust conclusions. This attrition rate is similar to data reported in comparable studies, ranging from around 16% [16] to a complete loss of patients [24]. The absence of information regarding systemic therapy in the study may result in significant data gaps, potentially exerting a substantial impact on the overall findings.

The retrospective nature of the study and the concomitant limitations in identifying patients receiving systemic therapy, coupled with the relatively modest sample size and substantial attrition during the follow-up, collectively underscore the intricacies and inherent constraints in the interpretation and generalizability of the study’s findings.

In summation, the study lacked tracking of the disease’s progression over time. Some research suggests that an early synovectomy might yield better outcomes, particularly in patients with RA.

## 5. Conclusions

In conclusion, our data substantiates a noteworthy recurrence-free survival rate in patients afflicted with chronic synovitis of the knee who underwent the combined treatment of synovectomy and RSO. This integrated approach emerges as an efficacious therapeutic intervention, markedly improving clinical symptoms. Nevertheless, additional investigations are imperative to scrutinize these hypotheses further. The imperative for conducting randomized and controlled trials is paramount, as they are essential for providing more robust evidence and corroborating these findings, thereby advancing our comprehension in this specific domain of study.

## Figures and Tables

**Table 1:** Demographic data.

Variable	PVNS *n* = 11 (30%)	RA *n* = 15 (40%)	Peripheral Spondyloarthritis *n* = 11 (30%)
**Age in years** mean (range)	34 (17–77)	38 (15–72)	32 (17–56)
**Sex** (male/female)	5/6	9/6	7/4
**BMI** mean (range)	24 (19–39)	26 (17–46)	24 (18–31)
**Operation** (Ask/open)	2/9	9/6	8/3
**Side** (right/left)	7/4	7/8	8/3
**Operation time in minutes** mean (range)	116 (55–235)	81 (30–165)	81 (25–150)
**Time interval between synovectomy and RSO** mean (range)	16 (6–46)	13 (5–47)	17 (5–54)
**Follow-up in months** mean (range)	70 (24–138)	82 (26–153)	100 (58–153)
**Oxford Knee Score** mean (range)	42 (37–48)	37 (12–47)	37 (26–47)
**Pain**	64%	73%	73%
**Swelling**	27%	47%	55%
**Re-synovectomy**	36%	33%	27%
**Re-synovectomy time in months** mean (range)	15 (4–21)	34 (14–64)	63 (48–90)

## Data Availability

Data is contained within the article.
